# Quality of life in Hidradenitis Suppurativa (Acne Inversa): A scoping review

**DOI:** 10.1002/ski2.214

**Published:** 2023-02-21

**Authors:** Amrit P. Kaur, Mary E. Laing, Laoise Griffin, Peter J. Carr

**Affiliations:** ^1^ Department of Dermatology University of Galway Galway Ireland

## Abstract

**Background:**

Hidradenitis Suppurativa (HS) is a chronic, relapsing, inflammatory skin condition which is physically, psychologically and socially disabling and often affects a patient's quality of life (QOL). There are numerous QOL tools used in dermatology. However, assessment of QOL in patients with HS is difficult due to the inability of generic QOL tools to specifically capture QOL in patients with HS. Numerous HS‐specific QOL tools have been developed in recent years. It is important to identify evidence on full psychometric evaluation of these tools.

**Objectives:**

There has been a gradual increase in the use of generic and disease‐specific QOL tools in the last few decades. The aim of this scoping review (SR) is to evaluate the most widely used generic QOL tools and HS‐specific QOL tools to identify the psychometric evaluation of such tools.

**Methods:**

Design: An SR guided by Joanna Briggs Institute manual and Arskey O’Malley framework guidelines. Data extraction included the studies available on full psychometric evaluation of the most widely used dermatology generic QOL tools in HS and HS‐specific QOL tools.

**Results:**

Ten papers were included in the review, eight papers demonstrated HS‐specific QOL assessment tools. The psychometric properties of these tools were underpinned by reliability, validity and sensitivity measurement. Six disease‐specific tools were identified in this SR. However, they all lack full psychometric evaluation.

**Conclusion:**

This review indicates that an extensive research in the field of QOL tools for HS is much needed. It is crucial to develop user‐friendly and validate disease‐specific tools to measure the real impact of disease on patients QOL. QOL instruments can evaluate the impact on life of an HS patient, thus helping improve intervention and management of disease. There is a necessity for more research into existing HS‐specific QOL tools and they should be widely tested and fully validated.

## Graphical abstract

Hidradenitis Suppurativa (HS) has debilitating effect on quality of life (QOL). Despite the various QOL tools, assessment of QOL in HS patients is difficult. This is likely owing to generic QOL instrument's inability to capture QOL in patients with HS. Therefore, the aim of this scoping review (SR) is to illuminate the importance of HS‐specific QOL tools. This SR identifies the lack of psychometric evaluation of the most widely used generic QOL tools in HS and HS‐specific QOL tools.

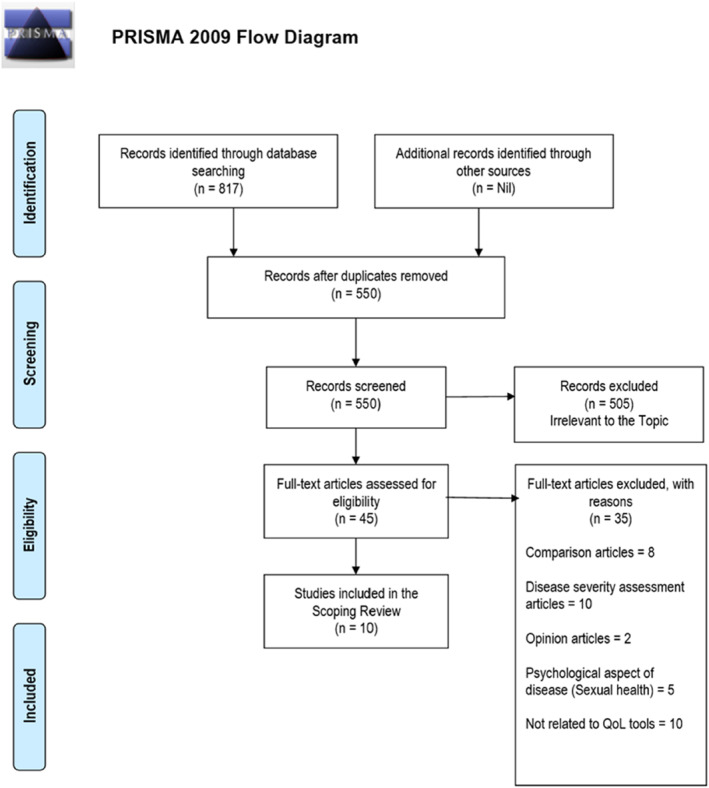

1

**What is already known about this topic?**

- Hidradenitis Suppurativa is a physically and psychologically disabling disease. It has a huge impact on quality of life (QOL) of a patient. There are generic and HS‐specific QOL tools to measure that impact.

**What does this study add?**

- It is a necessity to introduce more user‐friendly HS‐specific QOL tools with full psychometric evaluation as the ones available are not fully validated.

## 2 INTRODUCTION

Hidradenitis Suppurativa (HS) is an inflammatory condition that commonly affects the groin, buttocks, axillae and folds.^1^, ^2^ It is three times more common in females than in males and affects approximately ∼4% of the global population.^3^ European studies have suggested that HS may affect between 1% and 4% of the population, while a recent Irish study indicated a prevalence of 1.4%.^4^ Lesions of HS are associated with extreme pain, discharge and odour and therefore, can potentially lead to significant psychological impact and greatly affect quality of life (QOL).^5^ HS has a considerable negative effect on the life quality of persons who are affected by the disease as compared to other patients with other chronic skin conditions.^6^, ^7^ Patients' disease burden includes intense pain, work disability and thus leading to overall poor QOL. QOL is a multidimensional construct, which can be measured in various ways.^6^, ^7^ There are various QOL tools used in dermatology which can provide more in‐depth data. The principle of QOL tool is to identify the health state experienced by the subject by means of an appropriate questionnaire covering several different QOL‐related dimensions.^8^ Moreover, a global index (or several indices) is determined with calculations thus calculating QOL. The development of valid instruments to measure QOL in dermatology plays a vital role in assessing a patient and deciding on the treatment.^8^ Over the past years, QOL in patients with dermatological conditions has been broadly documented and various dermatology‐specific instruments have been described to measure this impact.^8^ There are some common generic QOL tools that are widely used in dermatology to assess QOL in patients with skin diseases including HS. For example, Dermatology Quality of life Index (DLQI) which was the first dermatology‐specific generic QOL instrument and to date is the most commonly used.^9^ However, generic or dermatological QOL measures may not capture changes in QOL particularly in HS.^5^ It's extremely important to identify if HS‐QOL tools are developed with psychometric properties such as reliability, validity and internal consistency measurements.^2^

### 2.1 Aims and objectives

The aim of this scoping review (SR) is to illuminate and describe the QOL tools used in HS patients, both general and specific, in terms of reliability, validity, sensitivity and internal consistency and to describe these psychometric properties to ensure changes in patients QOL are captured.

## 3 METHODS

**FIGURE 1:**
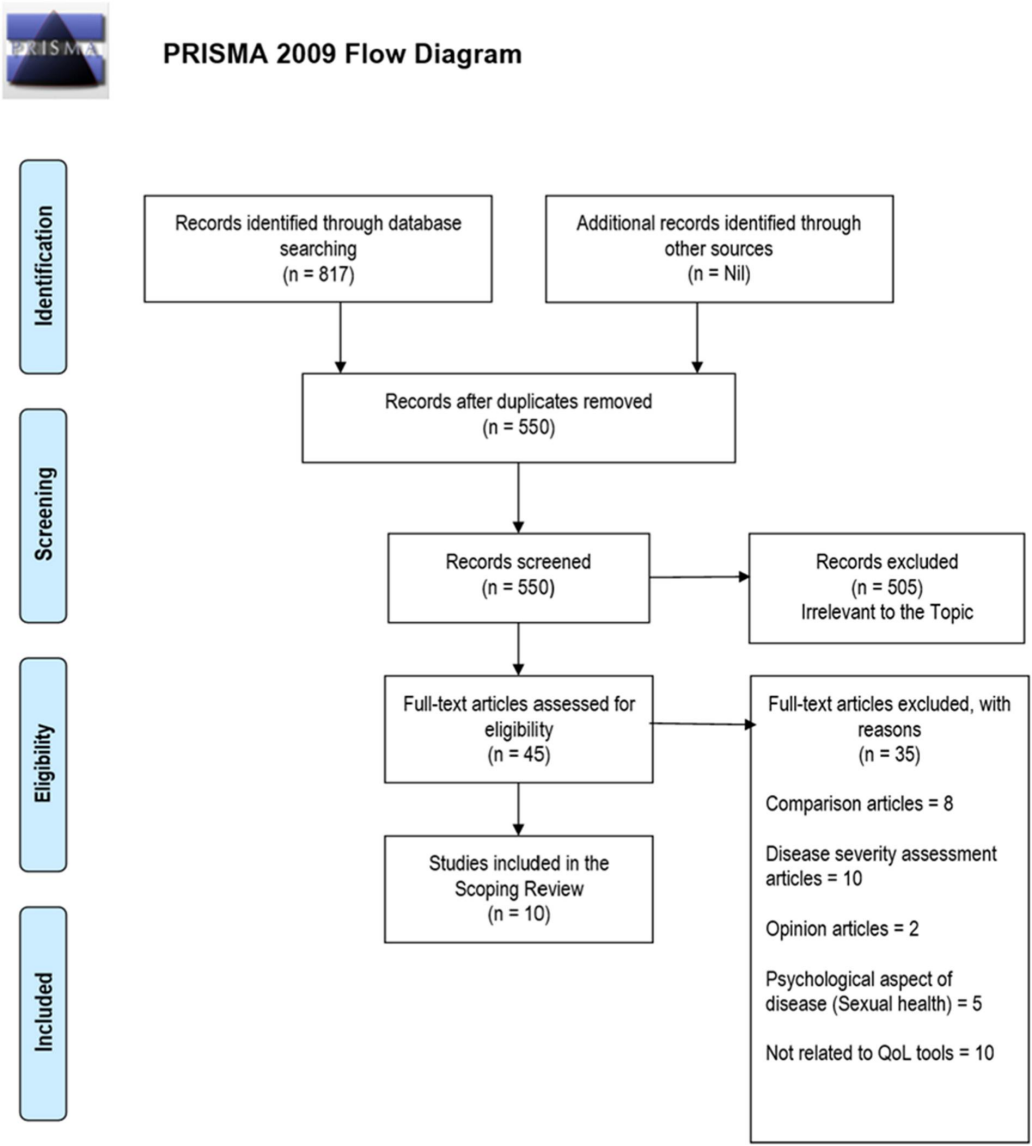
PRISMA flow diagram.

Research findings on the topic were summarized and disseminated, research gaps in the area were identified, recommendations for future research were made and the literature was mapped with relevance to time, location, source and origin. The methodology for this SR was guided by The Joanna Briggs Institute (JBI) guidelines^10^ and the preferred reporting system or framework for SR PRISMAs which has adapted guidance from Arksey & O’Malley framework.^11^ ^,^ ^12^ This SR research has been formulated using PCC (Population, Concept and Context) frame following a certain inclusion and exclusion criteria. Data extraction and synthesis were performed following the study selection using a template data extraction from JBI reviewer’s manual (Figure 1).^10^ Data are charted in the form of a table including study details, characteristics and extraction results (Table 1).^2^, ^3^, ^5^, ^6^, ^7^, ^9^, ^13^, ^20^ The review included identifying the research question, identifying relevant studies, study selection, charting the data and collating, summarizing and reporting the results.

**TABLE 1:** Data analysis of the studies included

Author	Type of study	Country	Context	Participants	QOL domains assessed	No. of items in the tool	Details of psychometric validation of the tool	External validation
Finlay and Khan^13^	Mixed method study	United Kingdom	Development of skin disease‐specific QOL tool for routine clinical practice. DLQI	One hundred and twenty patients with different skin conditions between the age of 15 and 70 years.	Symptoms and feelings Daily activities Leisure Work and school Personal relationships Treatment	10	*Reliability*: Test and retest reliability correlation coefficients obtained using Spearman correlation method. High correlation between overall DLQI scores noted (*ƴ*s = 0.99, *p* < 0.0001). The test‐retest reliability of individual questionnaires was high (*ƴ*s = 0.95–0.98, *p* < 0.001). *Internal consistency*: Consistency between all the questions in questionnaire was found to be significantly high, at the level of 0.002 and ranged from rank correlations of 0.23–0.70. Therefore, a high consistency. *Sensitivity*: A preliminary analysis was performed on first 60 patients and demonstrated reduction in the mean DLQI from 14.3 to 8.4, before and after admission, respectively.	No
Basra et al.^9^	Literature search/review and quantitative analysis	United Kingdom	Review of validation data of DLQI for skin disorders from 1994 to 2007	Two hundred and seventy‐two review articles	Symptoms and feelings Daily activities Leisure Work and school Personal relationship Treatment	10	*Reliability*: *Test‐retest reliability* of DLQI has correlation coefficient ranging from 0.56 to 0.99 and most studies showing values above 0.90. *Internal consistency reliability*: Expressed by Cronbach's alpha ranging from 0.75 to 0.92 which is considerably high. *Sensitivity*: DLQI has ability to detect change and adapt as patient's condition (for example before and after treatment). Thus highly sensitive to change and has high cross‐cultural validation value. *Validity*: *Construct*, *content and convergent validity* of DLQI is reported to be highly significant and has significant correlation with other generic and disease‐specific QOL instruments. DLQI is a validated QOL instrument for dermatology patients aged 16 years and above	No
Sisic et al.^14^	Mixed method	Canada and USA	Development of quality of life (QOL) instrument for HS and validation of this instrument.	Twenty‐one patients with HS (aged 18 years and older)	Twelve concepts: Impact on daily activities Symptoms due to HS Emotional consequences Restricted clothing content validity was demonstrated. But instrument is going through psychometric validation. Pending evaluation of reliability, discriminant capacity and responsiveness Psychosocial consequences Coping Sexual functioning Work and economic consequences Interactions with medical personnel Social support Symptoms due to treatment Concentration issues at work/leisure activities	53	*Content validity* was demonstrated. But instrument is going through psychometric validation. Pending evaluation of reliability, discriminant capacity and responsiveness.	No
			HS‐QOL					
Mclellan et al.^15^	Quantitative study (online collected data in the form of survey)	Canada	Preliminary psychometric evaluation of the HS‐QOL, assessing reliability and convergent validity	Fifty‐five adult patients (18–60 years) with HS from four dermatology clinics (one in Canada, one in Singapore and two in USA)	Seven domains. Physical consequences, HS symptoms, sexual activity consequences, emotional consequences, social consequences, work consequences, social support.	44	*Reliability*: *Internal consistency*: Cronbach's alpha value above 0.8 considered excellent. Seven subscales demonstrated excellent internal consistency, except for the social subscale which demonstrated adequate consistency. *Validity*: Measured in terms of correlation to other tools like DLQI and Dass‐21 and were in expected directions. Therefore, demonstrating HS‐QOL as a *valid instrument*. *Results*: HS‐QOL is a disease‐specific QOL assessment tool and indicated a strong evidence of reliability (internal consistency) and convergent validity.	No
Kirby et al.^2^	Mixed method design in four phases. Stage 1: Patient interviews. Stage 2: Cognitive debriefing interviews. Stage 3: Observational study of patients in USA. Stage 4: Observational study of patients in Denmark.	USA and Denmark	Validate HiSQOL instrument for clinical trials	Patients with HS aged 18 years and older	Symptoms Psychological Activities‐adaptations	17	*Reliability*: Excellent *internal consistency reliability* with Cronbach's alpha of 0.9 for HiSQOL *Test‐retest reliability* was also excellent for total HiSQOL scale. ICC of 0.90 and Cronbach's alpha of 0.94 *Validity*: *Convergent validity* confirmed (0.90) for HiSQOL as very strong correlation when compared to DLQI. *Further work required to confirm cross cultural validity*. HiSQOL has strong evidence for validity in assessing patient centred outcomes in clinical trials.	No
Pinard et al.^16^	Mixed method study	USA	Development of HSBOD (Hidradenitis Suppurativa Burden of Disease) tool to assess QOL in patients with HS Visual analog scale (VAS)	Twenty‐nine adult patients with HS.	Symptoms and feelings Daily activities Leisure Work/school Personal relationships	19	*Reliability*: *Reliability* assessed by Cronbach's alpha. HSBOD showed good internal consistency with Cronbach's alpha of 0.936 when compared to DLQI. Spearman rho was measured between HSBOD and DLQI as a measure of strength. There was a lack of strong correlation between two tools in terms of two domains which are personal relationships and work/school. Thus, representing shortcoming of DLQI in capturing many facets of HS. *Internal reliability* of HSBOD was shown and strongly correlated (p = 0.604, p < 0.001) with a validated QOL tool (DLQI) *Validity*: Due to small sample size further studies are requires to confirm the validity. Further studies with a broader data collection are required to confirm full psychometric evaluation.	No
Marron et al.^17^	Multicentre study with review of literature, interviews with group of patients, and then consensus of professionals from related health area	Spain	Development of new disease‐specific tools to assess QOL in HS and analysis of psychometric properties of this tool (HS‐QOL‐24)	Thirty adult patients with HS between the age of 18 and 61 years.	Psychosocial Economic Occupation Relationships Personal Clinical	24	*Reliability*: *Internal consistency*: (Cronbach alpha) 0.920 on initial evaluation (test) and 0.917 in the subsequent evaluation (retest) *Test‐retest reliability* : is good, measured using ICC (intra correlation coefficient) in a test‐retest intervention with most commonly used QOL tools in dermatology i.e., DLQI (Dermatology Quality of Life Index) and Skindex‐29. With ICC of 0.698 95% Cl (0.456–0.844) and 0.900 95% Cl (0.801–0.951), respectively. Validity: *Construct validity*: Assessed using a correlation and regression analysis with DLQI and Skindex‐29. Showed greater correlation values as compared to DLQI and Skindex‐29. Closer to 0.9 (0.863 and 0.898 for test and retest, respectively). *Cut points*: Delimited by comparing the range of values for HSQol‐24 and Skindex‐29 as latter yielded better results. *Sensitivity*: *Sensitivity* to change was assessed by calculating variations between HSQol‐24, DLQI and Skindex‐29. Correlation between change in HS‐QOL‐24 and Skindex‐29 observed as Pearson r = 0.504, p = 0.0004, respectively. And no statistically significant correlation with DLQI. *Results*: HS‐QOL‐24 is a disease‐specific instrument used to assess QOL in HS patients. It is reliable, valid and sensitive to change.	No
Aragones et al.^18^	Semi‐structured interviews of patients with HS and consensus between health professionals. Qualitative study and literature review	Spain	To validate and study a new screening tool (HS‐QOL‐24) to measure QOL in patients with HS.	One hundred and thirty adult patients with HS	Physical consequences HS symptoms Sexual activity consequences Emotional consequences Social consequences Work consequences Social	24	*Reliability*: *Internal consistency*: Cronbach alpha of 0.87, which indicates a good internal consistency. Excellent test‐retest reliability. *Validity and comparison*: The ICC (interclass correlation coefficient with the DLQI was 0.70 (*p* = 0.001) and 0.87 (*p* < 0.001) with the Skindex‐29 *Regression analysis*: Skindex showed 71% of variance and DLQI showed 49% of variance of HS‐QOL‐24 values. *Results*: HS‐QOL‐24 shows excellent properties as QOL instrument. It is comprehensible and has excellent reliability, validity.	No
Peris et al.^19^	Longitudinal study	Italy	Validation of new tool for assessment of HS burden HIDRAdisk	One hundred and forty adult patients with HS	Clinical Psychological Social	10	*Reliability*: *Internal consistency*: Raw Cronbach coefficient value was 0.894 and standardized Cronbach coefficient value was 0.898 which proves to be excellent. Correlation between the 10 items ranged between 0.308 and 0.673. *Test and retest reliability*: The spearman correlation coefficient for the *total hidradisk score was 0.8331. all icc results were >0.6* *Validity*: *Construct validity*: HIDRAdisk coefficient when compared with DLQI, Skindex 16, WPAI‐GH were statically significant (*p* < 0.0001). *Responsiveness to change* : Visit 1 to visit 3 were statistically significant for most parameters: Total HIDRAdisk score (*p* < 0.0001), which proves to be excellent. A visual validated tool that can improve management of HS	No
Thorlacius et al.^5^	Mixed method study (patients interviews, literature review, systematic search of studies, focus meetings and pilot testing) method	Denmark	Development of HS‐specific QOL instrument	Twenty‐one patients with HS aged 19–63 years in focus group interviews and 42 patients participated in pilot testing	Psychological Psychosocial Social Social job economy Physical Treatment Daily life with HS	23	No psychometric validation data available.	No
			HiSQOL				This QOL instrument proved to be acceptable, feasible and comprehensible when used in pilot testing.	

### 3.1 Literature search/inclusion and exclusion criteria

Comprehensive literature searches were conducted with EBSCO host (MEDLINE/PUBMED), CINAHL, PsychInfo, E‐journals) SCOPUS, Cochrane registry of controlled trials and Wiley online library. ProQuest and Greydoor were searched for any grey literature available on the topic. Key search terms included ‘HS’, ‘Hidradenitis Suppurativa’, ‘Acne Inversa’, ‘Quality of life’, ‘Quality of life Index’, ‘Quality of life Scale’, ‘Quality of life tools’, ‘disease severity’, ‘psychological aspect of HS’, disability, impact, burden, validation, validation of QOL tools in HS. All the databases were filtered by all times (No limitation), humans and English language.

Inclusion criteria:

1. Reviews with validation data available on most widely used generic dermatology QOL tool (DLQI) used in HS.
2. Studies on HS‐specific QOL tools developed till date
3. Only adult population with confirmed diagnosis of HS
4. Literature on psychometric evaluation studies of disease‐specific tools in HS

Exclusion criteria:

1. Paediatric population
2. Articles on drug therapy‐related QOL in HS
3. Studies not related to QOL in HS
4. Studies not related to psychometric evaluation or validation data in HS.

All the studies included in this SR are presented in the form of a Table (Table 1).

## 4 RESULTS

Our literature search returned 550 articles after the removal of duplicates. Following title and abstract screening, 505 were deemed not related to QOL in HS and were excluded. Excluded studies included comparison articles with evaluation between more than one QOL tool, articles focussed on sexual health and disease severity, opinion articles and those unrelated to QOL tools. Five articles were duplicate, not originally filed. Six were abstracts only. Five textbooks were excluded, 30 articles did not refer to QOL in HS and 40 articles were related to the treatment therapy in HS and improvement in QOL of patients with HS with different trials of medications. Eight articles were related to the paediatric population. Eighty‐three articles in total were excluded. The remaining 45 articles were included. Following the full text review, five articles were excluded, and 10 articles were included in this SR.

### 4.1 General description of included studies

Of 10 papers, three papers were published in the United Kingdom, three papers were published in Europe, two papers were published in the United States of America, and two in Canada. Studies and literature search were performed in the respective countries where they were published. The included papers are based on different types of studies. There were five mixed method studies, two quantitative studies with literature search, two qualitative studies with literature search and one longitudinal study.

### 4.2 Data analysis of the studies included

Basra et al.^9^ provide a comprehensive review of validation data and clinical results of DLQI between a period of 1994–2007,^13^, ^20^ identifying that DLQI has been used in 33 different skin conditions including HS. A detailed literature was conducted including 272 full text articles. Data concerning the development of DLQI from its development till 2007 were identified in terms of psychometric analysis. DLQI assessed in 12 different international studies using different correlation coefficients, ranged from 0.56 to 0.99 (with most studies showing values above 0.90, which is remarkably high. Internal consistency reliability of DLQI was assessed in 22 international studies using Cronbach's ɑlpha and ranged from 0.75 to 0.92. Study showed that DLQI has good responsiveness to change for most of the skin conditions (*p* < 0.0001); however, HS was not mentioned on list for sensitivity of DLQI. DLQI was used in a number of studies in parallel to other generic QOL instruments and showed good validity in terms of content and construct validity with a mean value of *r* = 0.58–0.78 and *p* < 0.0001). Overall, DLQI was found to be the most popular and most used generic QOL instrument in dermatology. However, an issue was raised regarding underrepresentation of emotional aspects of some skin conditions which are emotionally disabling, for example, HS. Although, wider ranges of dimensions are covered with generic measurement tools, many dimensions of HS in terms of QOL are not covered with DLQI. Therefore, HS‐specific scale is important for further research and disease management of HS. Another study by Jorge et al.^21^ indicates that DLQI exhibits adequate psychometric reliability and unidimensional structure for assessing QOL in Brazilian dermatology patients with Cronbach alpha result of 0.90 (Cl 95% 0.89–0.91), demonstrating that DLQI adequately assess the concrete discomforts of HS, however item performance varies in different sex and cultures and suffers from item bias which can be improved with disease‐specific questions.

A study by Sisic et al.^14^ developed an QOL instrument for HS (HS‐QOL). Patient interviews and expert opinion were used to develop a conceptual framework for HS. An HS‐QOL‐v1 measure was developed and pilot testing was done by conducting patient interviews, which resulted in HS‐QOL‐v2. It is a 53‐item questionnaire which is suitable for assessment of QOL in HS. According to the study, HS‐QOL‐v2 demonstrated content validity based on review of literature, concept elicitation interviews, item generation and cognitive interviews. The study indicated that psychometric validation is in process for evaluation of reliability, discriminant capacity and responsiveness. Mclellan et al.^15^ looked at the validation of HS‐QOL following their introduction of HS‐QOL instrument in 2017, which had 53 items. Fifty‐five adults with HS participated in the study. Participants completed 30‐min online survey. All the subscales indicated excellent reliability (internal consistency) except support subscale, which showed adequate consistency as compared to DLQI and DASS‐21 (Depression Anxiety Stress Scale) scores. It showed excellent validity as correlations with other measures were in expected direction. HS‐QOL was reduced to 44 items resulting in 7 subscale questionnaires. However, the study was limited by sample size; less number of items may increase the internal coefficiency of the items. Although, it requires further refinement and validation, it is an excellent instrument to assess QOL in patients with HS due to its multidimensional design.

Kirby et al.^2^ developed and validated another QOL instrument called Hidradenitis Suppurativa Quality of Life (HiSQOL) for clinical trial measurement of HS‐specific HR‐QOL (Health‐Related Quality of Life). A qualitative method was adopted in stage 1 conducting patients' interviews, stage 2 involved cognitive debriefing interviews and observational study of 222 patients was conducted in stage 3 which resulted in item reduction, validation measurement and psychometric properties assessment. Observational study of 215 patients was conducted in stage 4, to confirm the psychometric structure of the new scale. This 17 item QOL scale (HiSQOL) demonstrated reliability and validity to measure HS‐specific HR‐QOL in clinical trials. It showed excellent internal consistency reliability including each of the three subscales. However, responsiveness of the tool remains pending needing further research. Limitation to this study was that it under included the patients from different races and cultural beliefs. Moreover, further studies need to be performed for Differential Item Functioning analyses.

HSBOD (Hidradenitis Suppurativa Burden of Disease) is another HS‐specific QOL 2018 instrument developed in a mixed method study by Pinard et al.^16^ It is a 19‐item instrument which is self‐administered 10 cm visual analog scale (VAS) and was developed to understand the overall burden of disease in HS patients. HSBOD demonstrated strong internal consistency and convergent validity when compared to nondisease‐specific DLQI but lack the full psychometric analysis. The study is limited by small sample size and requires further studies to confirm selection of items, validity and reliability. Although, HSBOD yield good responses, it requires abstract thinking, which can be quite difficult for some patients due to the cultural background.

Another new disease‐specific questionnaire was developed by Marron et al.^17^ called HSQOL‐24. The study performed preliminary validation on the tool which indicated adequate reliability and validity values of the tool. Aragones et al.^18^ extended the psychometric evaluation of the tool at a later stage. The questionnaire was validated with a sample of 130 patients with HS. It is a first self‐administered QOL instrument in Spanish to assess QOL in patients with HS. This study demonstrated excellent reliability (test‐retest), internal consistency, validity and discriminative capacity of the instrument. The reliability study indicates strong internal accuracy and reproducibility with Cronbach's alpha of 0.920 (test) and 0.917 (retest). Intraclass correlation coefficient with DLQI and Skindex‐29 of 0.698 Cl 95% (0.456–0.844) and 0.900 Cl 95% (0.801–0.951), respectively.^17^, ^18^ For its use, cut‐off points were set, and the instrument was found to be prone to adjustment.

A study by Peris et al.^19^ demonstrated the psychometric evaluation of a new HS‐specific QOL tool known as HIDRAdisk. It is a VAS and composes 10 questions. Study demonstrated significant construct validity, excellent internal consistency reliability, good test‐retest reliability and good responsiveness to change. Therefore, a validated tool in Italian language that can improve management in HS. Although, it can be an effective QOL tool, limitation can be its need for translation into different foreign languages for different populations.

Thorlacius et al.^5^ developed another HS‐specific QOL tool called HiSQOL. A 23 item HS‐specific QOL questionnaire was developed. Study proved HiSQOL as comprehensible, acceptable and feasible which will prove quite beneficial in future to assess QOL in patients with HS. HiSQOL awaits psychometric evaluation. Limitations to this study analysis involve potential cultural bias with respect to the questionnaire's monocultural growth. This is therefore a possible weakness found in most processes of questionnaire growth. Furthermore, the study shows there could be a potential selection bias.^5^

Table 1 shows all the details/results extracted from included studies (in relation to the concept of the SR). Table 2 ^2^, ^3^, ^5^, ^6^, ^7^, ^9^, ^13^, ^20^ shows the psychometric properties of all the QOL tools identified in the review.

**TABLE 2:** Psychometric evaluation

Paper/Author	QOL tool	Reliability	Validity	Sensitivity	External validity
Finlay and Khan^13^	DLQI	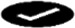	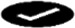	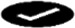	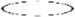
Basra et al.^9^	DLQI	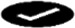	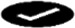	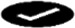	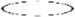
Sisic et al.^14^	HS‐QOL	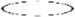	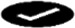	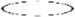	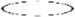
Mclellan et al.^15^	HS‐QOL	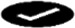	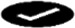	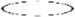	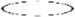
Kirby et al.^2^	HiSQOL	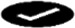	Needs to confirm cross cultural validity^a^	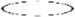	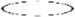
Pinard et al.^16^	HSBOD	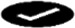	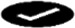	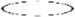	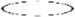
Marron et al.^17^	HS‐QOL‐24	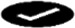	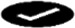	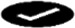	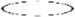
Aragones et al.^18^	HS‐QOL‐24	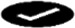	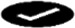	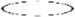	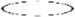
Peris et al.^19^	HIDRAdisk	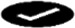	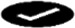	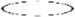	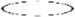
Thorlacius et al.^5^	HiSQOL	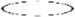	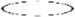	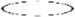	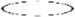

*Note*: Reliability: Measured in terms of reproducibility (test‐retest), which refers to similar outcomes from one study to another, if health status remains stable overtime. Validity: Refers to tools capacity to measure correctly for what it was built for. Sensitivity: Refers to a tool being able to detect any changes occurring in phenomenon of study. External validity: Refers to extent to which results from a study can be applied to other situations, events and groups.

^a^ Cross cultural validity refers to whether the measures originated in single culture are applicable and are equivalent in another culture.

## 5 DISCUSSION

This review has identified few studies measuring the impact of HS on QOL. Although, there are currently a limited numbers of HS‐specific QOL tools, they lack full psychometric evaluation (Table 2). None of these have previously been fully validated. There have been many generic QOL assessment tools used in patients with HS which could hardly capture the effect of disease on patients QOL. The features of HS as demonstrated by abscesses, fistulas and scarring have a big impact on physical and psychological functioning affecting QOL of patients.^5^ QOL measuring instruments which are disease specific can determine the factors inflecting QOL impact in HS compared to other diseases.

Although, DLQI is not HS‐specific QOL tool, it is the most widely used QOL assessment tool in dermatology. It has been the most popular and widely accepted tool over the year and is used in almost every skin condition including HS over the years. It is favoured by patients and dermatology practitioners. First developed by Finlay and Khan in 1994, it contains 10 questions which rate the intensity of dermatology affliction from 0 to 3, and two questions to evaluate the discomfort due to symptoms and their severity. Finlay and Khan^13^ assessed the reliability of DLQI and consistency of the tool for its clinical use in dermatology. Reliability and consistency and sensitivity of DLQI were found to be high according to the study by Basra et al.^9^ However, external validation remains pending. A good QOL instrument for HS needs to be developed with public patient involvement, face and content validity which should be piloted clinically before clinical trial testing.

HiSQOL by Kirby et al.^2^ is an acceptable QOL assessment tool, but cross‐cultural validity needs to be confirmed and its certain properties need to be elucidated. VAS like HSBOD and HIDRAdisk can be effective but some people may have difficulty with abstract thinking. HIDRAdisk needs to be translated into different languages and studied. There is no external validation available on any QOL tools for HS. The same conclusion applies to other tools that were identified in this SR. These are the clear research gaps evident in this SR. This review indicates that there is little research available in the field of QOL tools for HS as the disease can go undiagnosed or misdiagnosed for years and is not discussed as frequently as the other dermatological condition.

## 6 CONCLUSION

HS is a debilitating disease amongst other dermatological conditions with a remarkable effect on QOL. DLQI has been widely used to assess QOL in HS but HS has such a diverse experience on patients' life that it can hardly be measured by a generic questionnaire. Disease‐specific QOL instruments can evaluate real impact of the disease on QOL of an HS patient thus helping improved intervention and management of disease. All the QOL tools that are HS‐specific and developed recently are not fully validated except for HSQOL‐24 by Marron et al.^17^ In addition, no external validation was performed on any of the tools identified in this review. There has been some limited progress in understanding the psychosocial impact of the disease.^17^ In conclusion, there is a paucity of validated QOL tools for evaluation of QOL in HS. Measuring QOL impact should be a necessary objective of research and management of HS. There is a necessity for the further introduction of more user‐friendly QOL tools in HS and the existing HS‐specific QOL tools should be tested in a wider and more varied setting to assess its generalizability and assessed for full psychometric evaluation.

## CONFLICT OF INTEREST

None to declare.

## AUTHOR CONTRIBUTIONS

**Amrit P. Kaur**: Writing – original draft (Equal); Writing – review & editing (Equal). **Mary E. Laing**: Writing – review & editing (Supporting). **Laoise Griffin**: Writing – review & editing (Supporting). **Peter J. Carr**: Supervision (Supporting); Writing – review & editing (Supporting).

## ETHICS STATEMENT

Not applicable.

## Data Availability

Data sharing not applicable—no new data generated.
